# Pneumoproteins and biomarkers of inflammation and coagulation do not predict rapid lung function decline in people living with HIV

**DOI:** 10.1038/s41598-023-29739-x

**Published:** 2023-03-23

**Authors:** David M. MacDonald, Sarah Samorodnitsky, Chris H. Wendt, Jason V. Baker, Gary Collins, Monica Kruk, Eric F. Lock, Roger Paredes, Selvamuthu Poongulali, Danielle O. Weise, Alan Winston, Robin Wood, Ken M. Kunisaki, INSIGHT START Pulmonary Substudy Group, B. Aagaard, P. O. Jansson, M. T. Pearson, A. G. Babiker, A. Arenas-Pinto, N. B. Atako, E. Dennis, S. Forcat, F. Hudson, B. Jackson, D. Maas, C. Purvis, C. Russell, S. Emery, C. Carey, M. Clewett, S. Jacoby, F. Gordin, M. Vjecha, A. Sanchez, G. R. Loria, M. L. Doldan, A. Moricz, K. Tillmann, V. Müller, G. Touloumi, V. Gioukari, O. Anagnostou, P. Herrero, P. Lopez, A. Avihingsanon, P. Rerksirikul, E. Loiza, V. Mingrone, S. Lupo, F. Marconi, D. Daniel, A. Crinejo, M. French, L. Barba, D. Rowling, E. Warzywoda, M. Bloch, S. Agrawal, D. Dwyer, J. Taylor, L. van Petersen, L. Mertens, S. De Wit, K. Kabamba, M. Wolff, G. Allendes, M. Ristola, O. Debham, H. Jessen, A. Jessen, S. Wiebecke, H. Klinker, G. Fätkenheuer, C. Lehmann, I. Knaevelsrud, M. Rittweger, A. Stöhr, K. Olah, B. Schaaf, M. Hower, T. Harrer, E. Harrer, A. Skoutelis, V. Papastamopoulos, S. Metallidis, O. Tsachouridou, S. Pujari, A. Chitalikar, N. Kumarasamy, F. Beulah, E. Shahar, E. Kedem, D. Turner, J. Sierra Madero, C. Madrigal, K. M. El Filali, I. Erradey, E. Ekong, N. Eriobu, J. Valencia, M. León, E. Montalbán, J. Alave, R. Salazar, J. Vega, M. del Portal, F. Mendo, E. Bakowska, A. Ignatowska, M. Czarnecki, A. Szymczak, R. Wood, M. Rattley, S. Pillay, R. Mngqibisa, T. Ndaba, P. Madlala, V. Estrada, M. Rodrigo, M. Gutierrez, J. Muñoz, D. Dalmau, C. Badia, B. Clotet, J. M. Llibre, K. Ruxrungtham, S. Gatechompol, S. Kiertiburanakul, N. Sanmeema, C. Bowonwatanuwong, U. Ampunpong, W. Prasithsirikul, S. Thongyen, P. Chetchotisakd, S. Anunnatsiri, W. Ratanasuwan, P. Werarak, C. Kityo, H. Mugerwa, P. Munderi, J. Lutaakome, A. Clarke, A. Bexley, S. Das, A. Sahota, C. Emerson, S. McKernan, M. A. Johnson, M. Youle, J. Ross, J. Harding, S. Kegg, T. Moussaoui, F. Chen, S. Lynch, A. de Burgh-Thomas, I. Karunaratne, D. Dockrell, C. Bowman, A. Winston, B. Mora-Peris, D. R. Chadwick, P. Lambert, N. Desai, W. Carter, K. Henry, R. Givot, M. Chow, B. Holloway, S. Weis, I. Vecino, R. Novak, G. Culbert, A. Wilkin, L. Mosley, N. Thielman, J. Granholm, V. Watson, C. Clark, J. Santana, I. Boneta, I. Brar, L. Makohon, R. MacArthur, M. Farrough, M. Frank, S. Parker, E. Tedaldi, M. Santiago, S. Koletar, H. Harber, D. Thomas, I. Bica, B. Adams, C. Van Dam, M. Kolber, K. Moreno, A. Brown, B. Wade

**Affiliations:** 1 Minneapolis Veterans Affairs Health Care System, Pulmonary, Critical Care, and Sleep Apnea (111N), One Veterans Drive, Minneapolis, MN 55417 USA; 2 University of Minnesota, Minneapolis, USA; 3 Hennepin Healthcare Research Institute, Minneapolis, USA; 4 Hospital German Trias, Badalona, Spain; 5 Chennai Antiviral Research and Treatment Centre Clinical Research Site, CART-CRS-Infectious Diseases Medical Centre, VHS Chennai, Chennai, India; 6 Imperial College London, London, UK; 7 St. Mary’s Hospital, London, UK; 8 Desmond Tutu Health Foundation, Cape Town, South Africa; 9 University of Copenhagen, Copenhagen, Denmark; 10 University College London, London, UK; 11 University of New South Wales, Sydney, Australia; 12 Washington DC VAMC, Washington, District of Columbia, Washington, USA; 13 Fundación IBIS, Buenos Aires, Argentina; 14 University Hospital, Frankfurt, Germany; 15 National Kapodistrial University of Athens, Athens, Greece; 16 Spanish SSC, Acoiba, Madrid, Spain; 17 University Hospital, Bangkok, Thailand; 18 The HIV The Netherlands Australia Thailand Research Collaboration, Bangkok, Thailand; 19 Fundación IDEAA, Buenos Aires, Argentina; 20 Instituto Centralizado de Assistencia E Investigación Clínica Integral (CAICI), Rosario, Argentina; 21 Hospital Rawson, Córdoba, Argentina; 22 Royal Perth Hospital, Perth, Australia; 23 Sexual Health and HIV Service-Clinic 2, Brisbane, Australia; 24 Holdsworth House Medical Practice, Darlinghurst, Australia; 25 Westmead Hospital, Westmead, Australia; 26 Institute of Tropical Medicine, Antwerp, Belgium; 27 Centre Hospitalier Universitaire St. Pierre, St. Pierre, Belgium; 28 Fundación Arriarán, Santiago, Chile; 29 Department of Infectious Diseases, Helsinki University Central Hospital, Helsinki, Finland; 30 Gemeinschaftspraxis Jessen-Jessen-Stein, Berlin, Germany; 31 Universitätsklinikum Würzburg, Medizinische Klinik Und Poliklinik II, Schwerpunkt Infektiologie CRS, Wuerzburg, Germany; 32 Klinik I für Innere Medizin der Universität zu Köln, Studienbüro für Infektiologie u. HIV, Cologne, Germany; 33 EPIMED-Gesellschaft für Epidemiologische und Klinische Forschung in der Medizin GmbH, Berlin, Germany; 34 Ifi-Studien und Projekte GmbH, Hamburg, Germany; 35 Klinikum Dortmund gGmbH, Dortmund, Germany; 36 Universitätsklinikum Erlangen, Erlangen, Germany; 37 Evangelismos General Hospital, Athens, Greece; 38 AHEPA University Hospital, Thessaloniki, Greece; 39 Institute of Infectious Diseases, Kasauli, India; 40 YRGCARE Medical Centre VHS, Chennai CRS, Chennai, India; 41 Rambam Medical Center, Haifa, Israel; 42 Tel Aviv Sourasky Medical Center, Tel Aviv, Israel; 43 Instituto Nacional de Ciencias Médicas y Nutrición Salvador Zubirán (INCMNSZ), Mexico, Mexico; 44 University Hospital Centre Ibn Rochd, Casablanca, Morocco; 45 Institute of Human Virology-Nigeria (IHVN), Abuja, Nigeria; 46 Asociación Civil Impacta Salud y Educación, Barranco, Peru; 47 Asociación Civil Impacta Salud y Educacion-Sede San Miguel, Barranco, Peru; 48 Hospital Nacional Guillermo Almenara Irigoyen, Lima, Peru; 49 Hospital Nacional Edgardo Rebagliati Martins, Lima, Peru; 50 Wojewodzki Szpital Zakazny, Warsaw, Poland; 51 EMC Instytut Medyczny SA, Wrocław, Poland; 52 Desmond Tutu HIV Foundation Clinical Trials Unit, Cape Town, South Africa; 53 Durban International Clinical Research Site, Durban, South Africa; 54 Durban International Clinical Research Site (WWH), Durban, South Africa; 55 Hospital Clínico San Carlos, Madrid, Spain; 56 Hospital de la Santa Creu i Sant Pau, Barcelona, Spain; 57 Hospital Universitari Mutua Terrassa, Terrassa, Spain; 58 Hospital Universitari Germans Trias i Pujol, Barcelona, Spain; 59 Chulalongkorn University Hospital, Bangkok, Thailand; 60 Ramathibodi Hospital, Bangkok, Thailand; 61 Chonburi Regional Hospital, Ban Suan, Thailand; 62 Bamrasnaradura Infections Diseases Institute, Nonthaburi, Thailand; 63 Khon Kaen University, Srinagarind Hospital, Khon Kaen, Thailand; 64 Siriraj Hospital, Bangkok, Thailand; 65 Joint Clinical Research Center (JCRC), Kampala, Uganda; 66 MRC/UVRI Research Unit On AIDS, Entebbe, Uganda; 67 Brighton and Sussex University Hospitals NHS Trust, Brighton, UK; 68 Coventry and Warwickshire NHS Partnership Trust, Coventry, UK; 69 Belfast Health and Social Care Trust (RVH), Belfast, UK; 70 Royal Free London NHS Foundation Trust, London, UK; 71 University Hospital Birmingham NHS Foundation Trust, Birmingham, UK; 72 Lewisham and Greenwich NHS Trust, Greenwich, UK; 73 Royal Berkshire Hospital, Reading, UK; 74 Gloucestershire Royal Hospital, Gloucester, UK; 75 Sheffield Teaching Hospital NHS Foundation Trust, Sheffield, UK; 76 Imperial College Healthcare NHS Trust, London, UK; 77 The James Cook University Hospital, Middlesbrough, UK; 78 Florida Department of Health in Orange County/Sunshine Care Center, Orlando, USA; 79 Hennepin County Medical Center, Minneapolis, USA; 80 Hillsborough County Health Department/University of South Florida, Tampa, USA; 81 University of North Texas Health Science Center, Fort Worth, USA; 82 University of Illinois at Chicago, Chicago, USA; 83 Wake Forest University Health Sciences, Winston-Salem, USA; 84 Duke University Health System, Durham, USA; 85 Virginia Commonwealth University, Richmond, USA; 86 Puerto Rico-AIDS Clinical Trials Unit, Puerto Rico, USA; 87 Henry Ford Health System, Detroit, USA; 88 Newland Immunology Center of Excellence, Southfield, USA; 89 AIDS Resource Center of Wisconsin and Medical College of Wisconsin, Milwaukee, USA; 90 Temple University, Philadelphia, USA; 91 The Ohio State University Wexner Medical Center, Columbus, USA; 92 Washington DC VA Medical Center, Washington, DC USA; 93 Boston Medical Center, Boston, USA; 94 Regional Center for Infectious Disease, Greensboro, USA; 95 University of Miami, Coral Gables, USA; 96 Infectious Diseases Associates NW FL, PA, North Palm Beach, USA

**Keywords:** Predictive markers, Chronic obstructive pulmonary disease, HIV infections

## Abstract

Chronic obstructive pulmonary disease (COPD) is among the leading causes of death worldwide and HIV is an independent risk factor for the development of COPD. However, the etiology of this increased risk and means to identify persons with HIV (PWH) at highest risk for COPD have remained elusive. Biomarkers may reveal etiologic pathways and allow better COPD risk stratification. We performed a matched case:control study of PWH in the Strategic Timing of Antiretoviral Treatment (START) pulmonary substudy. Cases had rapid lung function decline (> 40 mL/year FEV_1_ decline) and controls had stable lung function (+ 20 to − 20 mL/year). The analysis was performed in two distinct groups: (1) those who were virally suppressed for at least 6 months and (2) those with untreated HIV (from the START deferred treatment arm). We used linear mixed effects models to test the relationship between case:control status and blood concentrations of pneumoproteins (surfactant protein-D and club cell secretory protein), and biomarkers of inflammation (IL-6 and hsCRP) and coagulation (d-dimer and fibrinogen); concentrations were measured within ± 6 months of first included spirometry. We included an interaction with treatment group (untreated HIV vs viral suppression) to test if associations varied by treatment group. This analysis included 77 matched case:control pairs in the virally suppressed batch, and 42 matched case:control pairs in the untreated HIV batch (n = 238 total) who were followed for a median of 3 years. Median (IQR) CD4 + count was lowest in the controls with untreated HIV at 674 (580, 838). We found no significant associations between case:control status and pneumoprotein or biomarker concentrations in either virally suppressed or untreated PWH. In this cohort of relatively young, recently diagnosed PWH, concentrations of pneumoproteins and biomarkers of inflammation and coagulation were not associated with subsequent rapid lung function decline.

**Trial registration:** NCT00867048 and NCT01797367.

## Introduction

Chronic obstructive pulmonary disease (COPD) and human immunodeficiency virus (HIV) are among the leading causes of death worldwide^1^. COPD is common in HIV, and HIV is an independent risk factor for the development of COPD, airflow obstruction, and worse lung function^2,3^. Decreased lung function, airway obstruction, and lower diffusing capacity are associated with increased mortality in people with HIV (PWH)^4,5^. Smoking, high viral load, and low nadir CD4 + T-cell counts increase susceptibility to COPD among PWH, but underlying mechanisms of how HIV affects COPD pathogenesis remain unknown and biomarkers of COPD risk in PWH have not been established^6–9^.

Biomarkers of systemic inflammation and coagulation in PWH are predictive of a variety of clinically important outcomes including death, cardiovascular disease, and malignancies^10,11^. Biomarkers of inflammation, coagulation, immune activation, and endothelial activation have been associated with cross-sectional lung function, but cross-sectional analyses are limited by the possibility of reverse causality, where a pulmonary process leads to an increase in biomarkers, whereas longitudinal analyses have the potential to predict lung function decline, and identify etiologic pathways^12–22^. Reliable predictors of lung function decline have been elusive, but higher blood concentrations of the pneumoprotein club cell secretory protein (CCSP) may be associated with slower decline in FEV_1_ in people with COPD and in the general non-HIV population^23,24^. The pneumoprotein surfactant protein D (SPD) has not been associated with longitudinal lung function in non-HIV COPD, but blocks HIV entry into target cells, is involved in innate immunity, and is reduced with initiation of antiretroviral therapy (ART); thus it may have a unique role in HIV-related lung disease^24–26^. The association of CCSP or SPD with longitudinal lung function decline in PWH has not been evaluated.

We sought to test the ability of the pneumoproteins SPD and CCSP, biomarkers of inflammation [C-reactive protein (CRP) and interleukin-6 (IL6)], and biomarkers of coagulation (d-dimer and fibrinogen) to predict lung function decline, both among PWH with effective viral suppression and those with untreated HIV.

## Methods

We conducted this study using data and stored plasma samples collected in the Strategic Timing of Antiretroviral Treatment (START) Pulmonary Substudy^27^.

### Parent cohort description

The design, methods, participant characteristics, and primary results of the parent START trial and its Pulmonary Substudy have been previously published^27–30^. Briefly, START enrolled HIV-positive, ART-naïve adults > 18 years of age with CD4 + T-cell counts > 500 cells/mm^3^ and then randomized participants to either immediate initiation of ART or deferred initiation until the CD4 + count declined to 350 cells/mm^3^ or AIDS developed. Participants were seen at study centers every 4 months, at which time fasting plasma was also collected for future analyses in those who provided informed consent for sample storage. Of 4685 participants enrolled in the parent START trial (from 215 sites in 35 countries), we co-enrolled 1026 participants (from 80 sites in 20 countries) into the START Pulmonary Substudy prior to randomization. In addition to the entry criteria for the parent START trial, additional Pulmonary Substudy criteria included the requirement that participants be ≥ 25 years old and free of factors affecting validity or safety of post-bronchodilator spirometry testing (e.g. respiratory illness within six weeks; surgery of the chest, abdomen, or eyes within three months; allergy to albuterol/salbutamol; unstable cardiac condition).

All START Pulmonary Substudy participants and START parent study participants provided informed consent specific to their study participation, all site institutional review boards/ethics committees approved the substudy, and we registered the substudy at ClinicalTrials.gov [NCT01797367 (2/22/2013) and NCT00867048 (2/23/2009)]. This study was performed in accordance with the relevant guidelines and regulations/ethical principles of the Declaration of Helsinki.

### Spirometry methods and outcomes

Study participants performed post-bronchodilator spirometry at baseline prior to randomization and annually during follow-up. Spirometry was performed using the EasyOne ultrasonic flow device (ndd Medical, Zurich, Switzerland) following inhalation of 180 mcg of albuterol/salbutamol via a metered dose inhaler. Author KMK centrally reviewed all spirometry tests for quality control. Repeat testing was requested when tests failed to meet published quality standards^31^. We used Global Lung Function Initiative (GLI) 2012 normative equations to determine predicted FEV_1_ and the lower limit of normal (fifth percentile) of the FEV_1_/FVC ratio^32^. FEV_1_ slope was determined using repeated measures mixed models in the original analysis and in this analysis^27^.

### Study design

We designed this analysis as a matched case:control study based upon rate of FEV_1_ decline. FEV_1_ decreases with age, but faster decline in FEV_1_ is a marker of COPD susceptibility and increased mortality^33–35^. Cases were defined as participants with rapid lung function decline, defined as an FEV_1_ slope of faster than − 40 mL/year. Though there is no consensus definition of rapid lung function decline, a rate of decline in FEV_1_ greater than 40 mL/year is commonly used^24,33,35^. Controls were defined as participants with stable lung function decline, defined as an FEV_1_ slope between − 20 and + 20 mL/year. We sought to test biomarkers of rapid lung function decline in two distinct groups of PWH: (1) those on ART with effective viral suppression and (2) those with untreated HIV. Therefore case:control identification was carried out in two distinct groups.

In the first group of case:control identification, we searched for matched case:control pairs among a virally suppressed group. In START, all participants were naïve to ART at study entry, so viral suppression could only be achieved at follow-up visits. Elite controllers, defined as having entered the study with HIV-RNA < 200 copies/mL despite lack of any previous ART exposure were excluded from this analysis. Participants could enter the viral suppression group if the met the following four criteria: (1) a minimum of 6 months with HIV-RNA < 200 copies/mL while on ART, (2) no subsequent HIV-RNA levels ≥ 200 copies/mL, (3) at least three high-quality annual FEV_1_ measurements beginning after at least 6 months of HIV-RNA < 200 copies/mL and (4) a corresponding plasma sample was available in the repository within ± 6 months of the first high-quality spirometry measure (and after the required 6 months with HIV-RNA level < 200 copies/mL). We allowed those in either arm of START (immediate or deferred ART initiation) to enter this analysis if they met these criteria, to better reflect clinical practice and allow a mix of CD4 + T-cell counts at the time of the plasma sample. This overall selection criteria allowed us to evaluate the relationship between biomarker concentrations and subsequent lung function decline in PWH who have successfully achieved viral suppression. We matched cases and controls (1:1 ratio) on sex and assigned randomization arm (immediate vs. deferred ART initiation), along with age (± 10 years), smoking status, and lung function (± 15% of predicted FEV_1_) at the first lung function test used in the analysis.

For the second case:control group, we searched for matched case:control pairs among an untreated HIV group. In START, since approximately half of participants were randomized to begin ART immediately after randomization, the untreated HIV group was restricted to those randomized to the deferred ART strategy. Participants could enter the untreated HIV group if they had three or more high-quality annual FEV_1_ measurements, no current or previous ART exposure at the time of those measurements, and a plasma sample available in the repository within ± 6 months of the first high-quality spirometry measurement. We also excluded elite controllers from this untreated HIV group. Due to the smaller pool of eligible participants in this untreated HIV analysis, we matched (1:1 ratio) on only age (± 10 years), smoking status, and lung function (± 15% of predicted FEV_1_) at the first lung function test used in the analysis.

### Biomarker measurements

Samples were collected at baseline and all follow up visits (1 month, 4 months, and every 4 months thereafter) and stored in a central laboratory at – 70 °C. Assays were performed using commercially available ELISA kits (Abcam, Cambridge, UK), with antibody kit numbers listed as [abxxxxxx] below. Participant samples were randomly assigned to individual masterplates and all ELISA assays were performed in duplicate with values below the limit of detection assigned the lower limit of detection value.

Our primary focus was to test the ability of the plasma pneumoproteins CCSP [ab238266] and SPD [ab239431] to identify risk of rapid lung function decline among PWH. We also investigated biomarkers with previously published data from PWH suggesting their potential to predict pulmonary outcomes in HIV-positive persons. These included the marker of Th1 inflammation interleukin-6 (IL-6 [ab178013]), the general systemic inflammatory response marker high-sensitivity C-reactive protein (hsCRP [ab181416]) and markers of activated coagulation (fibrinogen [ab208036] and d-dimer [ab196269]).

### Statistical analysis

We created histograms for each protein to assess skewness, and data were log transformed to improve normality if indicated. Pairwise concordance of replicate samples was assessed via Pearson’s correlation coefficient, and the log replicates were then averaged as the primary outcome variable in our models.

In our primary analyses we used standard linear models and linear mixed effects models to test whether case:control status and treatment status (virally suppressed vs untreated HIV) had a significant effect on the measured protein concentrations. We used these methods, rather than logistic regression, because one of our primary goals was to separately test associations in well controlled HIV and untreated HIV. Since matching was performed separately in each treatment group, there would be no association between treatment group and case:control status in logistic regression models. Logistic regression would also not have allowed us to test for an interaction between case:control status and treatment group on the levels of each biomarker. We hypothesized that the association between case:control status and biomarker concentrations may differ in the virally suppressed and untreated HIV groups, so we included an interaction term between case:control and treatment status (case:control × treatment status). We were unable to match for sex in the untreated HIV group, so sex was a covariate in those analyses. We also considered including a random effect for each matched pair, and a random intercept for the masterplate. We used the Akaike information criteria (AIC) to compare models of different complexity^36^. If the AICs for two models were within 2 units of each other, we selected the simpler model. We reported the significance of case:control status, treatment group, and their interaction on biomarker levels using Type I sums of squares based on the model selected using the AIC.

In secondary analyses we used paired t-tests to test whether log transformed biomarker concentrations varied by case:control status and treatment group. This was chosen as a secondary analysis as this method is straightforward but does not allow us to control for masterplate or sex, and additionally does not account for treatment group. We further used two-sample t-tests to test whether log-transformed biomarker concentrations varied by case:control status.

All statistical analyses were conducted using R version 3.6.1.

### Ethics declarations

The INSIGHT Network used the University of Minnesota Institutional Review Board (ID number: 0603M83587) for the START parent study and START Pulmonary Substudy. The START Pulmonary substudy was also approved by the institutional review board/ethics committee at each site.

### Study drugs

Antiretroviral drugs were donated to the central drug repository by AbbVie, Bristol-Myers Squibb, Gilead Sciences, GlaxoSmithKline/ViiV Healthcare, Janssen Scientific Affairs, and Merck.

## Results

### Participants and matching

**Figure 1:**
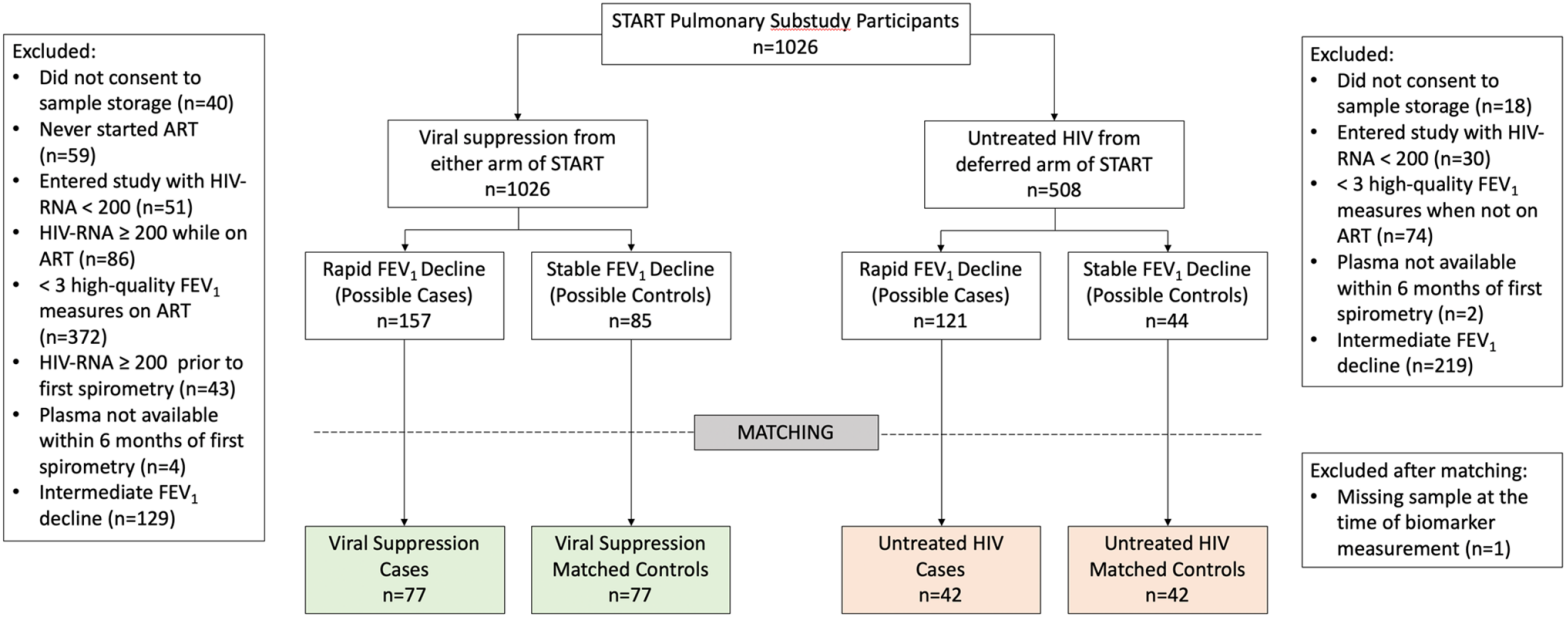
STROBE participant selection figure.

Of 1026 participants in the START Pulmonary Subsudy, we identified 77 eligible matched case:control pairs (n = 144 total) amongst the viral suppression group. We identified 43 matched pairs in the untreated HIV group, but at the time of biomarker measurement one serum sample was missing, and that case:control pair was excluded, leaving 42 matched pairs (n = 84 total) in the untreated HIV group (see Fig. 1 for STROBE figure). The most common reasons for exclusion were intermediate lung function decline (neither rapid nor stable, so neither meeting case or control criteria) and lacking three or more annual high-quality spirometry tests under the same treatment conditions (virally suppressed or untreated).

Characteristics of the cases and matched controls are shown in Table 1 for both the virally suppressed and untreated HIV groups. Median age was similar in cases and controls, across both groups. In the untreated HIV group, there were more female, and fewer Black participants among controls than among cases. Very few participants (n = 4 total) likely acquired HIV through injection drug use. As expected, CD4 + T cell counts at the first FEV_1_ measurement used were lower in the untreated HIV group than the virally suppressed group, but were similar in cases and matched controls in both groups. Median lung function, as measured by FEV_1_ percent predicted at the time of first included FEV_1_ measurement, was normal. There were minimal differences in lung function, HIV lab values, or timing of biomarker measurement between cases and matched controls in the viral suppression group, or the untreated HIV group (Supplementary Table S1). Longitudinal lung function decline in cases and matched controls for both groups is shown in Table 2. FEV_1_ decline was much faster in cases (− 86 and − 105 mL/year in virally suppressed and untreated, respectively) than controls (− 3 and − 5 mL/year in virally suppressed and untreated, respectively).

**Table 1:** Demographic and clinical characteristics of study participants. Data shown as median (interquartile range) or n (%). Data are reported at time of plasma biomarker assessment.

	Viral suppression		Untreated HIV	
	Cases n = 77	Controls n = 77	Cases n = 42	Controls n = 42
Demographics				
Age (years)	36.0 (31.0, 44.0)	35.0 (31.0, 41.0)	36.0 (29.8, 44.5)	37.0 (29.3, 42.0)
Female	16 (20.8%)	16 (20.8%)	16 (38.1%)	22 (52.4%)
Race				
Black	23 (29.9%)	19 (24.7%)	28 (66.7%)	14 (33.3%)
Latino/Hispanic	7 (9.1%)	20 (26.0%)	5 (11.9%)	8 (19.0%)
Asian	23 (29.9%)	10 (13.0%)	1 (2.4%)	7 (16.7%)
White	24 (31.2%)	28 (36.4%)	8 (19.0%)	12 (28.6%)
Other	0 (0.0%)	0 (0.0%)	0 (0.0%)	1 (2.4%)
Region				
Africa	21 (27.3%)	17 (22.1%)	25 (59.5%)	14 (33.3%)
Asia	23 (29.9%)	10 (13.0%)	1 (2.4%)	7 (16.7%)
Europe/Israel/Australia	15 (19.5%)	23 (29.9%)	3 (7.1%)	10 (23.8%)
Latin America	10 (13.0%)	20 (26.0%)	11 (26.2%)	10 (23.8%)
United States	8 (10.4%)	7 (9.1%)	2 (4.8%)	1 (2.4%)
HIV history				
Likely mode of HIV infection				
Injection drug use	1 (1.3%)	1 (1.3%)	0 (0.0%)	0 (0.0%)
Male sexual contact with person of same sex	47 (61.0%)	51 (66.2%)	12 (28.6%)	15 (35.7%)
Sexual contact with person of opposite sex	27 (35.1%)	23 (29.9%)	30 (71.4%)	24 (57.1%)
Other/unknown	2 (2.6%)	2 (2.6%)	0 (0.0%)	3 (7.1%)
Years HIV positive	0.8 (0.3, 3.0)	0.9 (0.3, 2.1)	1.6 (0.5, 3.4)	2.1 (0.8, 5.5)
HIV lab data				
CD4 + T cell count (cells/mm^3^)	747 (621, 947)	747 (628, 880)	722 (583, 831)	674 (580, 838)
CD8 + T cell count (cells/mm^3^)	806 (628, 925)	723 (573, 963)	926 (745, 1115)	961 (709, 1288)
CD4 + /CD8 + ratio	0.99 (0.76, 1.24)	1.01 (0.73, 1.31)	0.77 (0.55, 1.05)	0.74 (0.51, 0.90)
HIV-RNA (log_10_ copies/ml)	1.3 (1.3, 1.6)	1.3 (1.3, 1.6)	3.8 (3.1, 4.3)	3.9 (3.4, 4.4)
Medical history at study entry				
Body mass index (kg/m^2^)	25.2 (21.2, 27.0)	24.1 (22.0, 27.3)	25.5 (21.6, 27.4)	24.0 (21.8, 28.4)
Current smoker	19 (24.7%)	19 (24.7%)	11 (26.2%)	11 (26.2%)
Prior cardiovascular disease	0 (0%)	0 (0%)	0 (0%)	0 (0%)
Hypertension	15 (80.5%)	9 (11.7%)	10 (23.8%)	5 (11.9%)
Diabetes	1 (1.3%)	1 (1.3%)	0 (0%)	0 (0%)
Hepatitis B or C	4 (5.2%)	5 (6.5%)	1 (2.4%)	2 (4.8%)
Lung function, at first included measurement				
FEV_1_ (l)	3.54 (3.01, 4.01)	3.64 (2.85, 4.15)	3.15 (2.72, 3.58)	2.86 (2.24, 3.75)
FVC (l)	4.20 (3.54, 5.11)	4.44 (3.56, 5.05)	3.72 (3.26, 4.53)	3.53 (2.85, 4.71)
FEV_1_/FVC ratio	0.83 (0.80, 0.86)	0.82 (0.80, 0.85)	0.83 (0.79, 0.87)	0.84 (0.78, 0.88)
< 0.7	4 (5.2%)	0 (0%)	0 (0%)	4 (9.5%)
< Lower limit of normal^a^	4 (5.2%)	1 (1.3%)	1 (2.4%)	2 (4.8%)
FEV_1_ as % of predicted^1^	97.1 (86.4, 105.0)	95.6 (88.2, 102.9)	95.6 (89.8, 102.0)	95.7 (85.1, 107.7)

*FEV*_*1*_ forced expiratory volume in 1-s, *FVC* forced vital capacity, *HIV* human immunodeficiency virus, *START* strategic timing of antiretroviral therapy.

^a^From Global Lung Function Initiative (GLI) 2012 equations^32^.

**Table 2:** Longitudinal lung function in cases and controls by treatment group. Results are shown as median (interquartile range).

	Viral suppression		Untreated HIV	
	Cases n = 77	Controls n = 77	Cases n = 42	Controls n = 42
FEV_1_ slope (mL/year)	− 86.0 (− 125.0, − 65.0)	− 3.0 (− 11.4, 10.0)	− 105.0 (− 160.0, − 76.3)	− 5.0 (− 11.5, 3.5)
FVC slope (mL/year)	− 65.6 (− 96.6, − 14.7)	0 (− 32.3, 36.3)	− 61.0 (− 89.0, − 26.0)	− 5.0 (− 29.0, 22.0)
FEV_1_/FVC slope (% per year)	− 0.65 (− 1.16, − 0.04)	− 0.20 (− 0.57, 0.27)	− 0.53 (− 1.13, − 0.06)	− 0.45 (− 0.82, 0.14)
Number of FEV_1_ measurements	4 (3, 4)	4 (3, 5)	3 (3, 4)	4 (3, 4)

*FEV*_*1*_ forced expiratory volume in 1-s, *FVC* forced vital capacity, *mL* milliliters.

### Biomarkers

The correlation between experimental replicates was greater than 0.8 for all biomarkers considered. Histograms of the averaged biomarker replicates before and after log transformation are included in the online supplement (Supplementary Fig. S2).

### Associations between case:control status, treatment group, and biomarker concentrations

Results for our primary analysis, testing whether case:control status, treatment group (viral suppression vs untreated HIV), and their interaction affected biomarker concentrations, are shown in Table 3. Case:control status did not have a significant relationship with any of the six biomarkers. Treatment group had a significant relationship with d-dimer concentrations (F-value 6.49; p-value 0.01), but not other biomarkers. We also found a significant interaction between case:control status and treatment group on d-dimer concentrations (interaction p = 0.03), indicating that the association between case:control status and d-dimer concentrations varies by treatment group. However, the individual strata p-values were not statistically significant in either the virally suppressed (p = 0.49) or the untreated strata (p = 0.09).

**Table 3:** Effect of case:control status, treatment group, and their interaction (case:control status × treatment group) on biomarker concentrations. Cases represent participants with rapid FEV_1_ decline (≥ 40 mL/year), and controls are participants with stable FEV_1_ (− 20 mL/year to + 20 mL/year). Treatment group represents the effect of being in the virally suppressed vs untreated HIV groups on biomarker concentrations. All models incorporate sex as a covariate.

	F value	Pr (> F)
Surfactant protein D^a^		
Case:Control	0.24	0.62
Treatment group	0.08	0.78
Interaction	0.00	0.96
Club cell secretory protein		
Case:Control	0.69	0.41
Treatment group	0.35	0.56
Interaction	1.72	0.19
Interleukin-6^a^		
Case:Control	0.47	0.49
Treatment group	2.54	0.11
Interaction	0.25	0.62
C-reactive protein		
Case:Control	0.03	0.86
Treatment group	0.12	0.73
Interaction	1.34	0.25
Fibrinogen^a^		
Case:Control	2.02	0.16
Treatment group	0.009	0.92
Interaction	1.26	0.26
D-Dimer^a^		
Case:Control	0.47	0.49
Treatment group	6.49	0.01
Interaction	4.53	0.03

^a^These models incorporate a random effect for masterplate based on AIC values.

**Figure 2:**
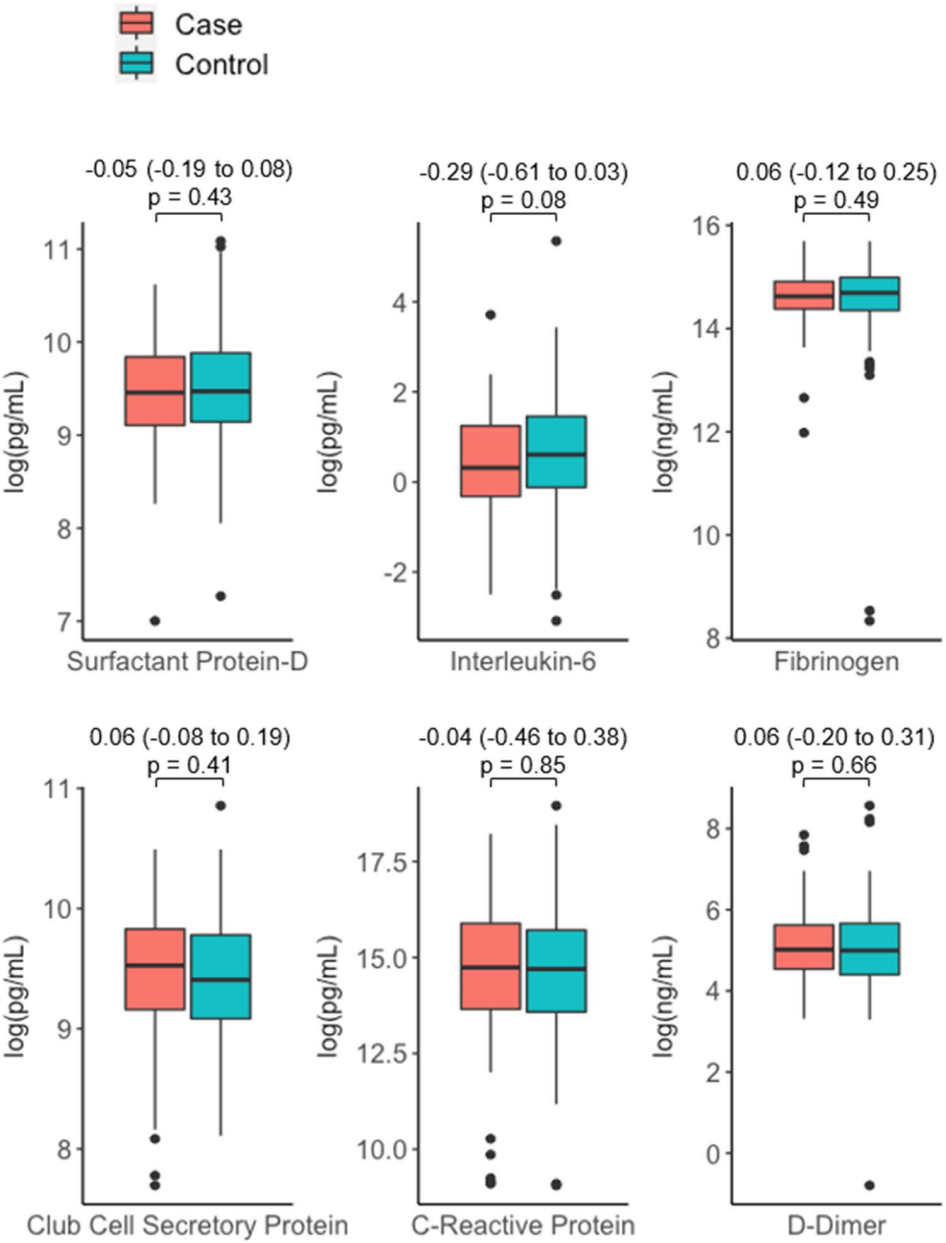
Comparison of log transformed biomarker concentrations by case:control status, where cases represent those with rapid lung function decline (FEV_1_ decline faster than 40 mL/year) and controls represent those with stable lung function (FEV_1_ change between − 20 and 20 mL/year). Values across the top represent the mean (95% confidence interval) of the differences in biomarker levels within each case control pair and p-values are from paired t-tests.

In secondary analyses comparing biomarker concentrations by t-testing rather than linear models, we found no significant difference in biomarker concentrations between cases and controls (Fig. 2). When comparing the untreated HIV and virally suppressed groups, both d-dimer concentrations [mean (95% CI) for difference in log (pg/mL) − 0.47 (− 0.76 to − 0.18)] and IL-6 concentrations [− 0.41 (− 0.75 to − 0.08)] were lower in the virally suppressed group (Supplementary Fig. S3).

## Discussion

We found no difference in plasma concentrations of pneumoproteins or biomarkers of inflammation and coagulation among PWH with rapid lung function decline compared to PWH with stable lung over a median of 3 years of follow up. Findings were consistent for both untreated HIV and virally suppressed groups.

The use of samples and data from the START Pulmonary Substudy uniquely allowed us to investigate associations between biomarkers and longitudinal lung function in two well characterized groups of PWH, with either untreated HIV or viral suppression. Based in large part on findings from the parent START trial, current recommendations are to immediately begin treatment for HIV at the time of diagnosis, but studying the untreated HIV group may still have relevance to persons who present late in the course of HIV infection or face barriers to accessing ART. The virally suppressed group represents current optimal care, with early ART initiation at high CD4 + T-cell counts. Findings among virally suppressed PWH have the potential to identify those most at risk for future lung disease and to reveal pathways important in HIV lung disease pathogenesis in the modern era of more widely available HIV testing and immediate treatment.

One of our primary goals was to evaluate the association between blood pneumoprotein concentrations and longitudinal lung function in PWH. Blood biomarkers of lung function decline have been elusive^37^. We measured two of the most well characterized blood pneumoproteins: (1) SPD, which is produced by type II alveolar cells, involved in innate immunity, and may have a unique role in HIV; and (2) CCSP, which is formed in the bronchioles, and is the only pneumoprotein that has shown a relationship with longitudinal lung function decline in multiple large studies^24,26,38–40^. Though lower CCSP concentrations may be predictive of faster lung function decline, these associations have been quite weak, even in large studies (r^2^ = 0.0043 for the relationship between log transformed CCSP concentrations and FEV_1_ decline in 4724 participants in the Lung Health Study, and a 1 SD decrease associated with a 4 ± 2.1 mL/year faster FEV_1_ decline in 2163 participants in ECLIPSE)^24,39^. We were likely underpowered to find a significant relationship between CCSP and rapid lung function decline. We also studied a cohort of PWH without established lung disease as opposed to these previous studies in non-HIV COPD, which included participants with at least moderate expiratory airflow obstruction. Though blood pneumoproteins are appealing because they can be collected rather simply, they cannot directly measure what is happening at the level of the lung tissue. Direct measurements of pneumoproteins from lung tissue or bronchoalveolar lavage may be more revealing, but these samples would require more invasive methods.

There are few previous analyses of blood pneumoproteins and lung function in PWH, and we are aware of no previous analyses of pneumoproteins and longitudinal lung function in PWH. In a cross-sectional analysis of PWH (n = 65) Jeon and colleagues found significant associations between higher CCSP and lower FVC, and higher CCSP and lower diffusing capacity for carbon monoxide (DL_CO_)^41^. Those findings were in contrast to general COPD as discussed above, where higher CCSP has been associated with better cross-sectional lung function and slower longitudinal lung function decline^24^. Analysis of CCSP in larger cohorts of PWH would help to clarify these associations and how they compare to non-HIV COPD, though the general population data suggest that CCSP may be a relatively weak marker of obstructive lung disease risk. Similar to our findings, Jeon et al. found no significant associations between SPD and lung function in PWH^41^.

We also found that treatment status (viral suppression or untreated HIV) had no effect on SPD or CCSP. These findings are consistent with Shiels and colleagues who found no significant differences in SPD concentrations between those on ART and not on ART^42^. We previously reported that SPD decreased after initiation of ART, but that was a small study (n = 15) in persons with more advanced HIV (median CD4 + cell count 320 cells/mm^3^), and the pathways leading to changes in SPD and lung function may be different in more advanced HIV^43^. Jambo and colleagues analyzed SPD concentrations in bronchoalveolar lavage (BAL) fluid from PWH and HIV negative participants. They found that BAL SPD concentrations were similar in HIV negative participants and PWH with CD4 + counts > 200, but SPD concentrations were greater in those with CD4 + counts < 200^44^. These data suggest that SPD may be more important in PWH with lower CD4 + counts, and though we analyzed a group of PWH with untreated HIV, very few participants reached CD4 + counts as low as these previous studies because ART was initiated at a CD4 + count of 350 cells/mm^3^_,_ and the study was terminated early at which time all participants were offered ART^28^.

We did not find a significant association between longitudinal lung function decline and biomarkers of inflammation and coagulation. There are few previous studies of these associations. Our findings are consistent with Gupte and colleagues who found that CRP had no association with longitudinal lung function in 619 South African PWH^15^. Our findings are also consistent with Fitzpatrick and colleagues who found that IL-6 was not associated with longitudinal lung function (n = 124 PWH in the Lung HIV study)^14^. Verboeket and colleagues recently found that higher hsCRP and lower IL-6 were associated with faster decline in FEV_1_, but as they discussed, these relationships may have been confounded by smoking (which is known to increase inflammatory markers and decrease lung function), and when they analyzed these relationships among non-smokers, they were no longer significant^22^. Lastly, in a previous analysis of the START Pulmonary Substudy utilizing biomarkers measured at the time of study entry (i.e. when all participants were ART naïve, but some were subsequently immediately initiated on ART), we found cross-sectional associations between biomarkers of inflammation and coagulation, but no associations with subsequent longitudinal lung function decline^18^. We are aware of no other data on the association between biomarkers of inflammation and coagulation and longitudinal lung function in PWH.

Biomarkers of inflammation and coagulation have been associated with cross-sectional lung function measures in previous studies of PWH. For example, in the study of 124 PWH in the Lung HIV study mentioned previously, higher IL-6 was associated with lower baseline FEV1% predicted and DL_CO_ % predicted, but not with longitudinal changes in either measure^14^. In a cross-sectional analysis of 147 HIV-positive individuals by the same authors, CRP and IL6 were associated with lower FEV1% predicted^13^. Cross-sectional analyses have not shown a relationship between fibrinogen and FEV_1_ in PWH, but higher fibrinogen may be associated with small airway dysfunction in PWH^16,18,20^ In our previous analysis of the START Pulmonary Substudy we found that IL-6, hsCRP, serum amyloid A (another inflammatory marker), and the IL-6/d-dimer score were associated with cross-sectional FEV_1_, but none associated with subsequent longitudinal lung function decline^18^. In sum, these data suggest reverse causality, where a pulmonary process leads to worse lung function, inflammation, and coagulation, as opposed to inflammation or dysregulated coagulation driving worsening lung function.

Though not the primary focus of this analysis, we also tested the effect of treatment group (virally suppressed vs untreated HIV) on biomarkers of inflammation and coagulation. In our primary analysis we found that d-dimer was lower among virally suppressed participants than among participants with untreated HIV; in secondary analysis, we found that both IL-6 and d-dimer concentrations were lower in virally suppressed participants than those with untreated HIV. This is consistent with a previous analysis in the Strategies for Management of Antiretroviral Therapy (SMART) trial where a randomized comparison of patients who received immediate ART showed a decrease in d-dimer and trend toward a decrease in IL-6, compared to participants who received deferred ART^45^. This is also consistent with a previous analysis of the parent START trial, in which participants assigned to immediate ART had significant reductions in d-dimer and IL-6 over 8 months compared to those assigned to deferred ART. There was no significant difference in change in hsCRP^46^.

Our study has several limitations. First, we did not measure other pneumoproteins, such as prosurfactant protein B, which have shown variation by HIV status and CD4 + counts^42^. We also did not measure markers of endothelial function, such as endothelin-1 which has been associated with longitudinal decline in FEV_1_ and DL_CO_, and macrophage activation, such as soluble CD163 which have been associated with longitudinal DL_CO_ decline (n = 70 PWH) 14]. Second, we did not have measures of diffusing capacity, which are the most frequently abnormal measure of lung function in PWH^43^. Third, we had a modest sample size which limited power to detect small effect sizes, particularly in the context of analyzing pneumoproteins which have shown very small effect sizes in non-HIV cohorts^24,39^. Fourth, we do not have bronchoalveolar lavage or other respiratory samples in the START Pulmonary Substudy, and findings in respiratory samples are not always reflected in plasma^47^. Lastly, we studied a cohort of generally young, recently diagnosed PWH with high CD4 + counts at relatively low risk of lung disease. Though this is a limitation, we feel it is also a strength, as this represents optimal care and should represent an increasing proportion of PWH in coming years. Though these participants were at a low risk of lung disease, the use of a matched case:control design allowed us to compare participants with rapid lung function decline (median FEV_1_ decline of 86 to 105 mL/year depending on group) to participants with stable lung function (median FEV_1_ decline 3 to 5 mL/year), and all participants had at least 3 high quality spirometry measures. This case:control design and large difference in lung function decline also maximized power given our modest sample size. Future work in our cohort will use untargeted metabolomic profiling methods to identify other potential biomarkers and pathways that might explain why these persons with early HIV experience such rapid lung function decline, independent of cigarette smoking.

Our study has several additional strengths. Our international, multi-center, clinical trial cohort is uniquely representative of the global HIV epidemic. FEV_1_ decline was the carefully standardized and quality controlled primary outcome of the substudy, thus providing high quality measures of lung function. We also utilized the randomization to immediate versus deferred ART to analyze associations in those with virally suppressed HIV and untreated HIV. Finally, this is the first study to analyze the association of pneumoproteins with longitudinal lung function decline among PWH.

In conclusion, we did not find an association between longitudinal FEV_1_ decline and blood concentrations of pneumoproteins or biomarkers of inflammation and coagulation in PWH. Future studies should explore other pathways and consider non-spirometry outcomes such as DL_CO_.

## Supplementary Information

Supplementary Information.

## Data Availability

Requests for data can be submitted to the START Scientific Steering Committee upon completion of START. The Research Proposal form can be found here: http://insight.ccbr.umn.edu/research_proposal/.
