# Supplementary material for: Pneumoproteins and biomarkers of inflammation and coagulation do not predict rapid lung function decline in people living with HIV
Source: Sci Rep. 2023 Mar 23;13:4749. doi: 10.1038/s41598-023-29739-x (PMC10036615; doi:10.1038/s41598-023-29739-x)
Supplement: Supplementary file 1 — Supplementary Information. [file 41598_2023_29739_MOESM1_ESM.pdf]

Online Data Supplement

Pneumoproteins and biomarkers of inflammation and coagulation do not predict rapid lung function decline in people living with HIV

Authors:
David M. MacDonald, MD, MS[1,2], Sarah Samorodnitsky[2], Chris H. Wendt, MD[1,2], Jason V. Baker, MD, MS[3,2], Gary Collins, MS[2], Monica Kruk[2], Eric F. Lock[2], Roger Paredes, MD, PhD[4], Selvamuthu Poongulali, PhD[5], Danielle O. Weise[2], Alan Winston, MD[6,7], Robin Wood, DSc [Med][8], and Ken M. Kunisaki, MD, MS[1,2] for the INSIGHT START Pulmonary Substudy Group[9]

1: Minneapolis Veterans Affairs Health Care System, Minneapolis, USA
2: University of Minnesota, Minneapolis, USA
3: Hennepin Healthcare Research Institute, Minneapolis, USA
4: Hospital German Trias, Badalona, Spain
5: Chennai Antiviral Research and Treatment Centre Clinical Research Site, CART-CRS-Infectious Diseases Medical Centre, VHS Chennai, India
6: Imperial College London, London, UK
7: St. Mary's Hospital, London, UK
8: Desmond Tutu Health Foundation, Cape Town, South Africa
9: Consortium representative is Ken M. Kunisaki[1,2]

Contents of Online Data Supplement

S1 Table: Differences in matched case:controls characteristics.

S2 Figure: Histograms of averaged replicates for each pneumoprotein and biomarker before and after log transformation.

S3 Figure: Comparison of biomarker concentrations by treatment status.

S4: Complete START Pulmonary Substudy group roster.

S1 Table: Differences in lung function, HIV lab values, and timing of biomarker measurement between cases (rapid lung FEV_1 decline) and controls (stable FEV_1 decline) in virally suppressed and untreated HIV groups. Differences are reported as median (IQR) of (cases – controls).

	**Viral suppression**	**Untreated HIV**
FEV_1 %predicted	1.6 (-5.8, 7.1)	1.0 (-4.0, 8.6)
FVC % predicted	0.1 (-5.3, 6.0)	1.2 (-4.9, 7.2)
FEV1/FVC ratio	0.39 (-3.55, 4.38)	-0.84 (-6.01, 5.95)
CD4+ T-cell count (cells/mm^3)	2.0 (-133.0, 160.0)	25.0 (-146.5, 193.0)
HIV-RNA (log_{10} copies/mL)	0.0 (-20.0, 1.0)	-338.0 (-16848, 28268)
Number of FEV_1 measurements	0.0 (-1.0, 1.0)	0.0 (-1.0, 0.0)
Days from ART initiation to biomarker assessment	-15.0 (-126.0, 50.0)	n/a
Days from HIV-RNA <200 for 6 months to biomarker assessment	-14.0 (-128.0, 24.0)	n/a
Days from biomarker assessment to first spirometry	0.0 (0.0, 116.0)	0.0 (0.0, 9.0)

ART, antiretroviral therapy; FEV_1, forced expiratory volume in 1-second; FVC, forced vital capacity; HIV, human immunodeficiency virus; IQR, interquartile range.

S2 Figure: Histograms of averaged replicates for each pneumoprotein and biomarker before and after log transformation.

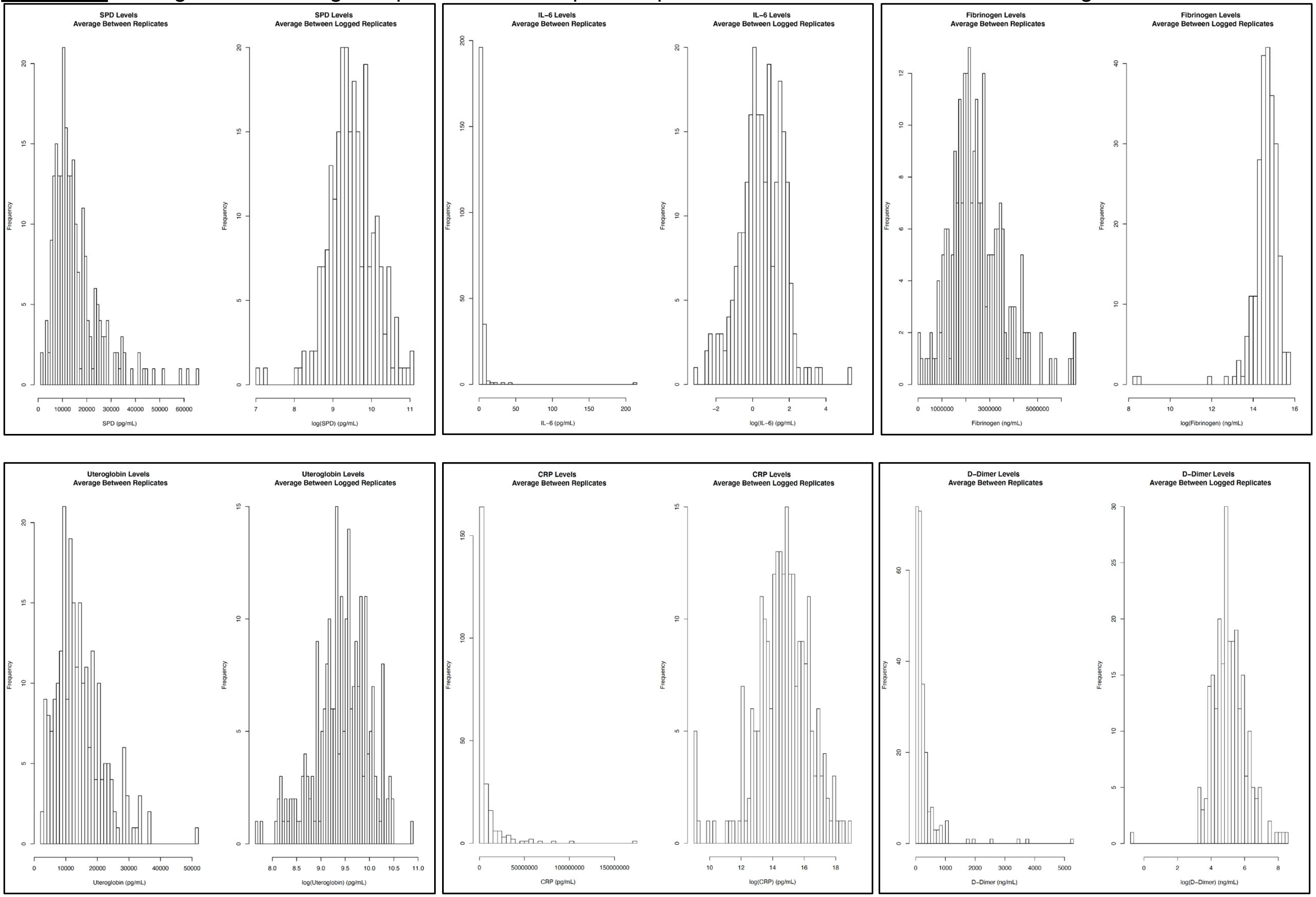

Twelve measures of CRP, 9 measures of D-dimer, and 4 measures of fibrinogen (out of a total of 456 measures of each) were below the lower limit of detection and were replaced with the lower limit of detection.

S3 Figure: Comparison of biomarker concentrations by treatment status (virally suppressed vs untreated HIV). Values across the top represent mean (95% confidence interval) of the difference in average biomarker levels in participants with viral suppression compared to participants with untreated HIV. P-values are from two-sample t-tests.

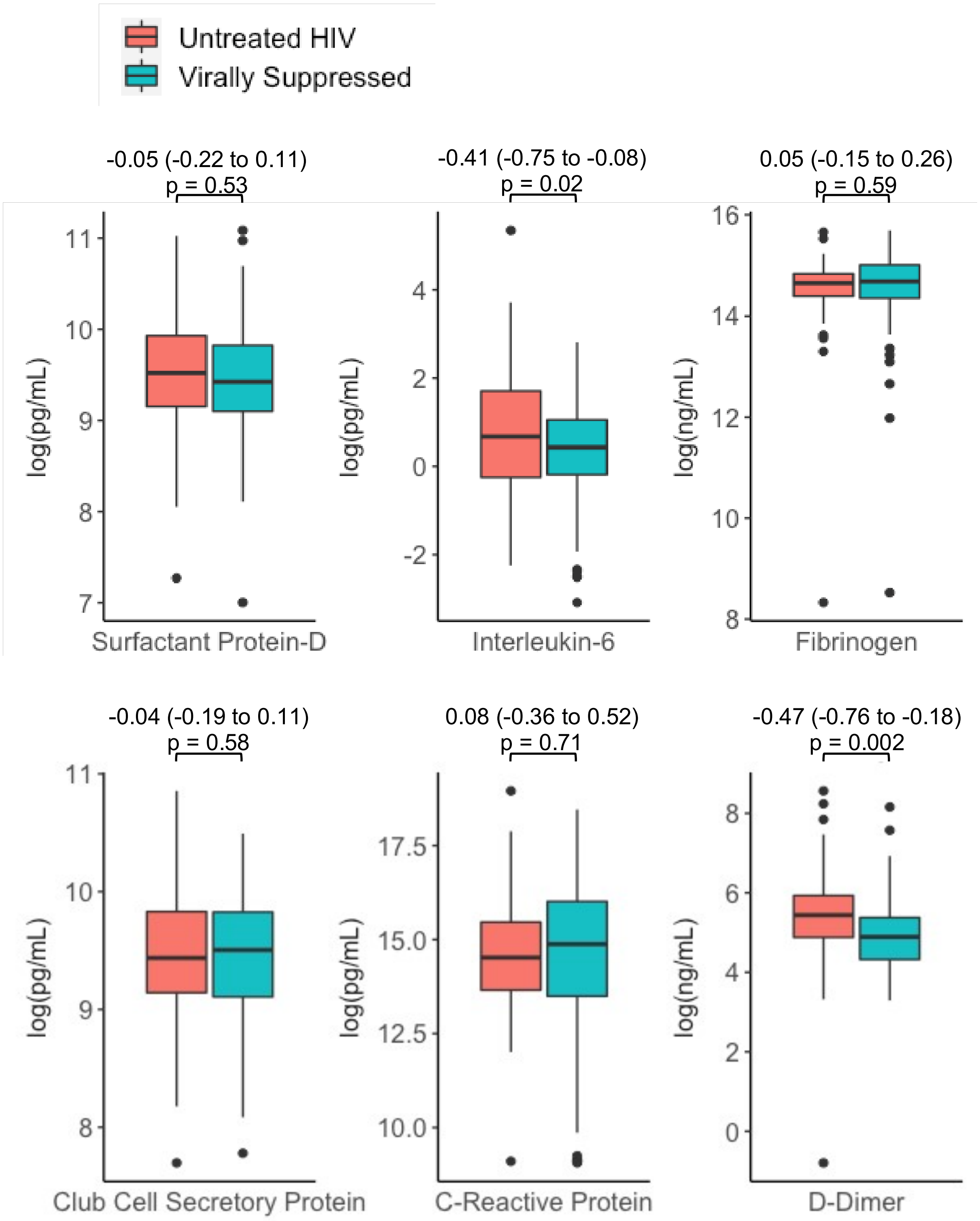

S4: START Pulmonary Substudy Group Roster.

International Coordinating Centres
Copenhagen (University of Copenhagen, Copenhagen, Denmark): B Aagaard, PO Jansson, MT Pearson.
London (University College London, London, UK): AG Babiker, A Arenas-Pinto, NB Atako, E Dennis, S Forcat, F Hudson, B Jackson, D Maas, C Purvis, C Russell.
Sydney (University of New South Wales, Sydney, Australia): S Emery, C Carey, M Clewett, S Jacoby.
Washington (Washington DC VAMC, Washington, District of Columbia, United States): F Gordin, M Vjecha, A Sanchez.

Site Coordinating Centres
Argentina (Fundación IBIS, Buenos Aires), Argentina: GR Loria, ML Doldan, A Moricz.
Germany (University Hospital, Frankfurt, Germany): K Tillmann, V Müller.
Greece (National Kapodistrial University of Athens, Athens, Greece): G Touloumi, V Gioukari, O Anagnostou.
Spain (Spanish SSC, Acoiba, Madrid, Spain): P Herrero, P Lopez.
Thailand: A Avihingsanon (University Hospital, Bangkok, Thailand), P Rerksirikul (The HIV Netherlands Australia Thailand Research Collaboration, Bangkok, Thailand).

Site Investigators by Country and institution

Argentina
Fundación IDEAA: E Loiza, V Mingrone.
Instituto Centralizado de Assistencia e Investigación Clínica Integral (CAICI): S Lupo, F Marconi.
Hospital Rawson: D Daniel, A Crinejo.

Australia
Royal Perth Hospital: M French, L Barba.
Sexual Health and HIV Service - Clinic 2: D Rowling, E Warzywoda.
Holdsworth House Medical Practice: M Bloch, S Agrawal.
Westmead Hospital: D Dwyer, J Taylor.

Belgium
Institute of Tropical Medicine: L van Petersen, L Mertens.
Centre Hospitalier Universitaire St. Pierre: S De Wit, K Kabamba.

Chile
Fundación Arriarán: M Wolff, G Allendes.

Finland
Helsinki University Central Hospital, Dept of Infectious Diseases: M Ristola, O Debham.

Germany
Gemeinschaftspraxis Jessen-Jessen-Stein: H Jessen, A Jessen.
Universitätsklinikum Würzburg, Medizinische Klinik und Poliklinik II, Schwerpunkt Infektiologie CRS: S Wiebecke, H Klinker.
Klinik I für Innere Medizin der Universität zu Köln, Studienbüro für Infektiologie u. HIV: G Fätkenheuer, C Lehmann.

EPIMED-Gesellschaft für epidemiologische und klinische Forschung in der Medizin GmbH: I Knaevelsrud, M Rittweger.
Ifi - Studien und Projekte GmbH: A Stöhr, K Olah.
Klinikum Dortmund gGmbH: B Schaaf, M Hower.
Universitätsklinikum Erlangen: T Harrer, E Harrer

Greece
Evangelismos General Hospital: A Skoutelis, V Papastamopoulos. AHEPA
University Hospital: S Metallidis, O Tsachouridou.

India
Institute of Infectious Diseases: S Pujari, A Chitalikar.
YRGCARE Medical Centre VHS, Chennai CRS: N Kumarasamy, F Beulah.

Israel
Rambam Medical Center: E Shahar, E Kedem.
Tel Aviv Sourasky Medical Center: D Turner.

Mexico
Instituto Nacional de Ciencias Médicas y Nutrición Salvador Zubirán (INCMNSZ): J Sierra Madero, C Madrigal.

Morocco
University Hospital Centre Ibn Rochd: KM El Filali, I Erradey.

Nigeria
Institute of Human Virology-Nigeria (IHVN): E Ekong, N Eriobu.

Peru
Asociación Civil Impacta Salud y Educación: J Valencia, M León.
Asociación Civil Impacta Salud y Educacion - Sede San Miguel: E Montalbán, J Alave.
Hospital Nacional Guillermo Almenara Irigoyen: R Salazar, J Vega.
Hospital Nacional Edgardo Rebagliati Martins: M del Portal, F Mendo.

Poland
Wojewodzki Szpital Zakazny: E Bakowska, A Ignatowska. EMC
Instytut Medyczny SA: M Czarnecki, A Szymczak.

South Africa
Desmond Tutu HIV Foundation Clinical Trials Unit: R Wood, M Rattley.
Durban International Clinical Research Site: S Pillay, R Mngqibisa.
Durban International Clinical Research Site (WWH): T Ndaba, P Madlala.

Spain
Hospital Clínico San Carlos: V Estrada, M Rodrigo.
Hospital de la Santa Creu i Sant Pau: M Gutierrez, J Muñoz.
Hospital Universitari Mutua Terrassa: D Dalmau, C Badia.
Hospital Universitari Germans Trias i Pujol: B Clotet, JM Llibre.

Thailand
Chulalongkorn University Hospital: K Ruxrungtham, S Gatechompol.
Ramathibodi Hospital: S Kiertiburanakul, N Sanmeema.

Chonburi Regional Hospital: C Bowonwatanuwong, U Ampunpong.
Bamrasnaradura Infections Diseases Institute: W Prasithsirikul, S Thongyen.
Khon Kaen University, Srinagarind Hospital: P Chetchotisakd, S Anunnatsiri.
Siriraj Hospital: W Ratanasuwan, P Werarak.

Uganda
Joint Clinical Research Center (JCRC): C Kityo, H Mugerwa.
MRC/UVRI Research Unit on AIDS: P Munderi, J Lutaakome.

United Kingdom
Brighton and Sussex University Hospitals NHS Trust: A Clarke, A Bexley.
Coventry and Warwickshire NHS Partnership Trust: S Das, A Sahota.
Belfast Health and Social Care Trust (RVH): C Emerson, S McKernan.
Royal Free London NHS Foundation Trust: MA Johnson, M Youle.
University Hospital Birmingham NHS Foundation Trust: J Ross, J Harding.
Lewisham and Greenwich NHS Trust: S Kegg, T Moussaoui.
Royal Berkshire Hospital: F Chen, S Lynch.
Gloucestershire Royal Hospital: A de Burgh-Thomas, I Karunaratne.
Sheffield Teaching Hospital NHS Foundation Trust: D Dockrell, C Bowman.
Imperial College Healthcare NHS Trust: A Winston, B Mora-Peris.
The James Cook University Hospital: DR Chadwick, P Lambert.

United States
Florida Department of Health in Orange County/Sunshine Care Center: N Desai, W Carter.
Hennepin County Medical Center: K Henry, R Givot.
Hillsborough County Health Department/University of South Florida: M Chow, B Holloway.
University of North Texas Health Science Center: S Weis, I Vecino.
University of Illinois at Chicago: R Novak, G Culbert.
Wake Forest University Health Sciences: A Wilkin, L Mosley.
Duke University Health System: N Thielman, J Granholm.
Virginia Commonwealth University: V Watson, C Clark.
Puerto Rico-AIDS Clinical Trials Unit: J Santana, I Boneta.
Henry Ford Health System: I Brar, L Makohon.
Newland Immunology Center of Excellence: R MacArthur, M Farrough.
AIDS Resource Center of Wisconsin and Medical College of Wisconsin: M Frank, S Parker.
Temple University: E Tedaldi, M Santiago.
The Ohio State University Wexner Medical Center: S Koletar, H Harber.
Washington DC VA Medical Center: D Thomas.
Boston Medical Center: I Bica, B Adams.
Regional Center for Infectious Disease: C Van Dam.
University of Miami: M Kolber, K Moreno.
Infectious Diseases Associates NW FL, PA: A Brown, B Wade.
